# Changes in spindle morphology driven by TPX2 overexpression in MYC-driven breast cancer cells

**DOI:** 10.17912/micropub.biology.001182

**Published:** 2024-04-10

**Authors:** Guadalupe Pena, Julia Rohrberg, Andrei Goga, Rebecca Heald

**Affiliations:** 1 Molecular and Cell Biology, University of California, Berkeley, Berkeley, California, United States; 2 Medicine, University of California, San Francisco, San Francisco, California, United States; 3 Cell & Tissue Biology, University of California, San Francisco, San Francisco, California, United States

## Abstract

The MYC oncogene was previously shown to induce mitotic spindle defects, chromosome instability, and reliance on the microtubule-associated protein TPX2 to survive, but how TPX2 levels affect spindle morphology in cancer cells has not previously been examined in detail. We show that breast cancer cell lines expressing high levels of MYC and TPX2 possess shorter spindles with increased TPX2 localization at spindle poles. A similar effect was observed in non-transformed human RPE-1 cells compared to a tumor cell line (HeLa) that overexpresses MYC . These results demonstrate that TPX2 alters spindle length and morphology in cancer cells, which may contribute their ability to divide despite MYC-induced mitotic stress.

**Figure 1. Higher TPX2 expression in MYC-driven breast cancer cell lines decreases spindle length and increases TPX2 intensity, especially at spindle poles:**
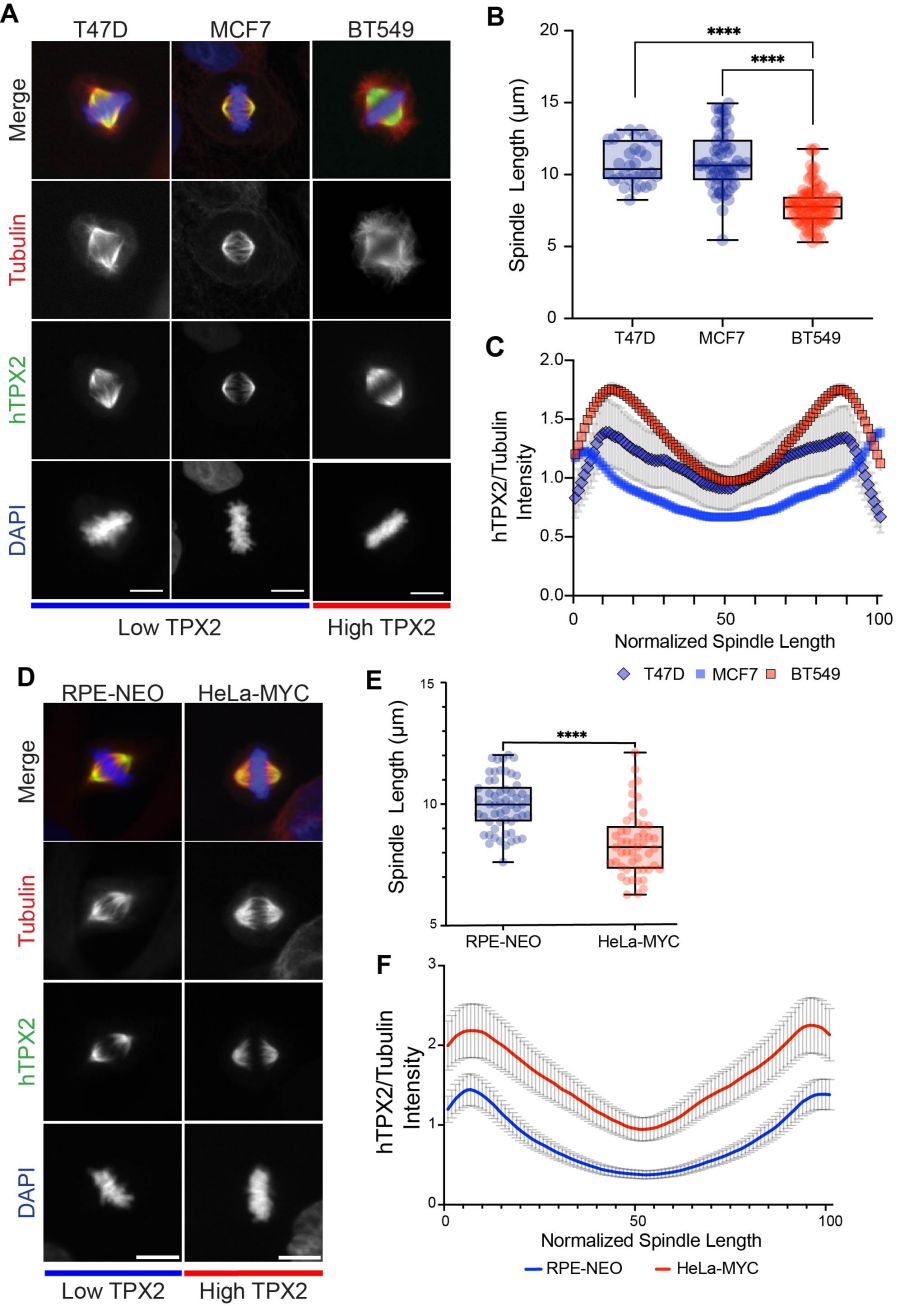
(A) Immunostaining of metaphase spindles from low and high TPX2 expressing breast cancer cell lines. Scale bar = 10 µm. (B) Quantification of spindle length, T47D n = 32, MCF7 n = 65, BT549 n = 95. Boxplot shows median marked at center and data maxima and minima indicated by whiskers. Box shows 25th to 75th percentiles. **** = P < 0.0001 from two-tailed unpaired t tests. (C) Line scan analysis of TPX2 /Tubulin fluorescence intensity, Mean ± SEM, T47D n = 25, LY2 n = 45, BT549 n = 115 (D) Immunostaining of fixed metaphase RPE-NEO and HeLa-MYC cells. (E) Quantification of spindle lengths, RPE-NEO n = 56, HeLa-MYC n = 50. Boxplot shows median marked at center and data maxima and minima indicated by whiskers. Box shows 25th to 75th percentiles, **** = P < 0.0001 from two-tailed unpaired t test. (F) Line scan analysis of TPX2 /Tubulin fluorescence intensity, Mean ± SEM, RPE-NEO n = 53, HeLa-MYC n = 52.

## Description

MYC is an oncogene that is overexpressed in many aggressive human cancers (Felsher & Bishop, 1999; Soucek & Evan, 2010) . It was shown previously that overexpression of MYC leads to error-prone mitosis and spindle assembly defects accompanied by up-regulation of several spindle-associated genes, including the microtubule-associated protein TPX2 (Rohrberg et al., 2020) . Targeting Protein for Xklp2 ( TPX2 ) is a RanGTP-regulated importin cargo that is overexpressed in many aggressive human cancers and is associated with chromosomal instability (Asteriti et al., 2010; Carter et al., 2006; Castro et al., 2007; Hu et al., 2012; Neumayer et al., 2014) . Interestingly, TPX2 depletion was shown to be synthetically lethal with MYC overexpression (Rohrberg et al., 2020) .

In a variety of systems, TPX2 has been shown to be essential for spindle bipolarity, microtubule nucleation, stabilization, and organization at spindle poles (Brunet et al., 2004; Schatz et al., 2003; Tulu et al., 2006) . TPX2 is indispensable for microtubule branching nucleation (Brunet et al., 2004; Petry et al., 2013) and also binds and activates the mitotic kinase Aurora A (Bayliss et al., 2003; Eyers et al., 2003; Tsai et al., 2003) . Using *Xenopus* egg extracts, we showed previously that addition of recombinant TPX2 results in significantly shorter spindles and a change in microtubule organization (Helmke & Heald, 2014) . However, it is poorly understood how overexpression of TPX2 affects spindle architecture in human cancer cells.

To examine the effect of TPX2 overexpression on spindle morphology, we analyzed three breast cancer cell lines with either high or low MYC and TPX2 levels ( Figure 1A ). Tubulin and TPX2 immunofluorescence of BT549 cells expressing high levels of MYC /TPX2 revealed significantly shorter spindles than the low MYC/TPX2 expressing lines T47D and MCF7. The high MYC/TPX2 cell line showed greater recruitment of TPX2 along the length of the spindle, with a greater accumulation at the spindle poles compared to the low MYC/TPX2 cell lines ( Figure 1B &C). We next compared a non-tumorigenic human retinal pigment epithelium (RPE-1) cell line versus Hela cells that overexpress MYC. Immunofluorescence and spindle length analysis again showed that cells expressing higher levels of TPX2 displayed significantly shorter spindles than the control ( Figure 1D &E). Similar to the high TPX2 expressing breast cancer cells, line scan analysis of the TPX2/tubulin intensity ratio showed that high MYC Hela cells recruited increased levels of TPX2 along the length of the spindle ( Figure 1F ).

In summary, this analysis revealed that cell lines with high levels of TPX2 and MYC possess morphologically distinct spindles compared to cells expressing low MYC / TPX2 . Future experiments could elucidate how TPX2 mediates this effect by recruiting other spindle factors such as Aurora A and/or by altering microtubule branching nucleation and organization. TPX2 upregulation and dependency across various aggressive cancers make it an attractive target for cancer therapies. Understanding how TPX2 contributes to spindle architecture could provide useful insight into how this linchpin spindle assembly protein protects genomically unstable cancer cells.

## Methods

**Cell culture**

BT549 and T47D breast cancer cell lines were grown in RPMI supplemented with 10% FBS, 10 U/ml penicillin and 10 mg/ml streptomycin at 37°C. MCF7 breast cancer cells, RPE-1 and Hela were grown in DMEM supplemented with 10% FBS and 10 U/ml penicillin, 10 mg/ml streptomycin at 37°C.

**Immunofluorescence**

Cells were seeded overnight on 12 mm coverslips, fixed for 2 minutes in with -20°C methanol in freezer, washed three times with 1x PBS and permeabilized with 0.5% Triton X-100 in 1x PBS for 5 minutes at room temperature. Cells were incubated with blocking buffer (1% goat serum, 0.1% Triton X-100, and 9.8 mg/ml of bovine serum albumin in 1x PBS) for 1 hour at room temperature. Primary antibodies were diluted with 3% BSA in 1x PBS and added to cells for 1 hour at room temperature. Cells were washed three times quickly followed by three 5 minutes washes of 1x PBS. Secondary antibodies were diluted with 3% BSA in 1x PBS and added to cells for 30 minutes at room temperature. Cells were washed three times quickly followed by three 5 minutes washes of 1x PBS. Final PBS wash contained 0.05 µg/ml of DAPI. Cells were washed two times quickly with 1x PBS before being mounted with ProLong Diamond reagent (Invitrogen). Listed antibodies were used as indicated: Rabbit anti-TPX2 (1:1000, HPA005487, Sigma), mouse anti-beta tubulin (E7; Developmental Studies Hybridoma Bank, Iowa City, IA), rabbit secondary antibody conjugated to Alexa Fluor 488 (1:500, A21206, Invitrogen), and mouse secondary antibody conjugated to Alexa Fluor 568 (1:500, A21124, Invitrogen).

**Microscopy**

Metaphase cells were imaged using micromanager software (Edelstein et al., 2014) with an Olympus BX51 microscope using an ORCA-ER camera (Hamamatsu Photonics), and with an Olympus UPlan FL 40× air objective.

**Quantification and Statistical Analysis**

Spindle length quantification: Individual spindle length measurements were made using Fiji. Pole to pole distances are represented in boxplots with the thick line indicating average length and upper and lower box boundaries indicating 75th and 25th percentiles, respectively. The minimum number of spindles measured (n) is listed in the figure legend. Statistical significance was determined by unpaired two sample t test using GraphPad Prism version 10.0.0 for MacOS, GraphPad Software, Boston, Massachusetts USA, www.graphpad.com . p values are listed in the figure legend.

Fluorescence intensity line scans quantification: Line scans were generated using an automated Java ImageJ plugin developed by X. Zhou ( https://github.com/XiaoMutt/AiSpindle , see Extended Data; Gibeaux et al., 2018). Line scans quantify the average ratio of h TPX2 to tubulin fluorescence intensity across the spindle length from pole to pole. Spindle length is normalized across the range from 1-100. The number of spindles measured in each condition (n) is listed in the figure legend. Error bars indicate ± standard error over the mean (SEM).

## Reagents

**Table:** 

**Name**	**Source**	**Catalog No.**
Rabbit anti-TPX2	Sigma	HPA005487
Mouse anti-beta tubulin	DSHB	E7
Alexa Fluor 488	Invitrogen	A21206
Alexa Fluor 568	Invitrogen	A21124

## Extended Data

Description: AiSpindle by Xiao Zhou. A Java ImageJ plugin for fluorescence intensity line scan quantification.. Resource Type: Software. DOI: 10.22002/a9kfq-rh826

## References

[R1] Asteriti IA, Rensen WM, Lindon C, Lavia P, Guarguaglini G (2010). The Aurora-A/TPX2 complex: a novel oncogenic holoenzyme?. Biochim Biophys Acta.

[R2] Bayliss R, Sardon T, Vernos I, Conti E (2003). Structural basis of Aurora-A activation by TPX2 at the mitotic spindle.. Mol Cell.

[R3] Brunet S, Sardon T, Zimmerman T, Wittmann T, Pepperkok R, Karsenti E, Vernos I (2004). Characterization of the TPX2 domains involved in microtubule nucleation and spindle assembly in Xenopus egg extracts.. Mol Biol Cell.

[R4] Carter SL, Eklund AC, Kohane IS, Harris LN, Szallasi Z (2006). A signature of chromosomal instability inferred from gene expression profiles predicts clinical outcome in multiple human cancers.. Nat Genet.

[R5] Edelstein AD, Tsuchida MA, Amodaj N, Pinkard H, Vale RD, Stuurman N (2014). Advanced methods of microscope control using μManager software.. J Biol Methods.

[R6] Eyers PA, Erikson E, Chen LG, Maller JL (2003). A novel mechanism for activation of the protein kinase Aurora A.. Curr Biol.

[R7] Felsher DW, Bishop JM (1999). Transient excess of MYC activity can elicit genomic instability and tumorigenesis.. Proc Natl Acad Sci U S A.

[R8] Gibeaux R, Acker R, Kitaoka M, Georgiou G, van Kruijsbergen I, Ford B, Marcotte EM, Nomura DK, Kwon T, Veenstra GJC, Heald R (2018). Paternal chromosome loss and metabolic crisis contribute to hybrid inviability in Xenopus.. Nature.

[R9] Helmke KJ, Heald R (2014). TPX2 levels modulate meiotic spindle size and architecture in Xenopus egg extracts.. J Cell Biol.

[R10] Hu Y, Wu G, Rusch M, Lukes L, Buetow KH, Zhang J, Hunter KW (2012). Integrated cross-species transcriptional network analysis of metastatic susceptibility.. Proc Natl Acad Sci U S A.

[R11] Neumayer G, Belzil C, Gruss OJ, Nguyen MD (2014). TPX2: of spindle assembly, DNA damage response, and cancer.. Cell Mol Life Sci.

[R12] Pérez de Castro I, de Cárcer G, Malumbres M (2007). A census of mitotic cancer genes: new insights into tumor cell biology and cancer therapy.. Carcinogenesis.

[R13] Petry S, Groen AC, Ishihara K, Mitchison TJ, Vale RD (2013). Branching microtubule nucleation in Xenopus egg extracts mediated by augmin and TPX2.. Cell.

[R14] Rohrberg J, Van de Mark D, Amouzgar M, Lee JV, Taileb M, Corella A, Kilinc S, Williams J, Jokisch ML, Camarda R, Balakrishnan S, Shankar R, Zhou A, Chang AN, Chen B, Rugo HS, Dumont S, Goga A (2020). MYC Dysregulates Mitosis, Revealing Cancer Vulnerabilities.. Cell Rep.

[R15] Schatz CA, Santarella R, Hoenger A, Karsenti E, Mattaj IW, Gruss OJ, Carazo-Salas RE (2003). Importin alpha-regulated nucleation of microtubules by TPX2.. EMBO J.

[R16] Soucek L, Evan GI (2009). The ups and downs of Myc biology.. Curr Opin Genet Dev.

[R17] Tsai MY, Wiese C, Cao K, Martin O, Donovan P, Ruderman J, Prigent C, Zheng Y (2003). A Ran signalling pathway mediated by the mitotic kinase Aurora A in spindle assembly.. Nat Cell Biol.

[R18] Tulu US, Fagerstrom C, Ferenz NP, Wadsworth P (2006). Molecular requirements for kinetochore-associated microtubule formation in mammalian cells.. Curr Biol.

